# The place of free will and agency in psychiatric practice: Commentary on … William James and British thought: then and now

**DOI:** 10.1192/bjb.2019.89

**Published:** 2020-04

**Authors:** Steve Pearce

**Affiliations:** Oxfordshire Complex Needs Service, Oxford Health NHS Foundation Trust, UK

**Keywords:** Ethics, history of psychiatry, personality disorders, philosophy, consent and capacity

## Abstract

In psychiatric practice, professionals tend to split patients into those who are responsible for their actions, and those who are not. This approach does a disservice to both groups. Patients assumed to retain agency may be blamed, and those assumed to lack agency are disempowered. Professionals should adopt a more nuanced approach to agency and control, recognising that it is impaired in most psychiatric disorders, but absent in very few. This is possible without making stigma worse.

## William James’ views on free will

The article by Leary^1^ encourages a reappraisal of William James's contribution to the early study of psychology. He is best known for his interest in, and lectures on, the psychology of religious experience,^2^ but Leary notes another central interest, in free will. He notes James's doubts in his early writings that even ‘a wiggle of the will’ is possible. James writes, for example, that addicts (‘dipsomaniacs’) experience a compulsion^a^ unlike anything experienced by other people, an impulse that is irresistible (James 1890, quoted in Pickard^3^).The presence or absence of responsible agency, meaning the ability to make choices and act upon them (thus an expression of free will), is central to modern psychiatric practice, and one of the ways in which James' interests continue to be relevant.

## Agency as an all-or-nothing concept

Interest in agency by mental health professionals has fluctuated. Ideas such as locus of control and generalised self-efficacy have been the subject of research, but refer to the person's beliefs about their ability to choose and act, rather than their ability to make choices and carry through the actions associated with them (agency). In psychiatry, agency itself has been neglected as a subject for study,^4^ although in practice it is generally assumed either to be present or absent. In other words mental health workers, like their peers in the rest of society, have a tendency to split patients into those who have agency – who have the freedom to choose to act differently – and those who do not, and are powerless to control their behaviour.

The tendency to dichotomise agency in this way is not restricted to psychiatry. The legal system treats people as either responsible for their actions (having *mens rea*) or not, a dichotomy echoed in the choice between two possible verdicts.^5^ Addiction provides a recent example of the way agency is dichotomised in mental health. Before the 20th century addictions were generally considered character flaws, consisting of unwise choices freely made. The adoption of the illness model of addiction (a movement with a long history of which James was a part, crystallising in 1939 with the birth of Alcoholics Anonymous^6^) alters the formulation to that of someone who is unable to resist their impulses to use, latterly formulated as owing to changes in brain chemistry.^7^ Thus agency is either retained, along with fault, or lost, which allows for compassionate treatment. For a discussion of the debate as it pertains to addictions, see Pickard^3^ and Pickard and Pearce.^8^

## Agency in psychiatry

The tendency to dichotomise agency in psychiatry leads to patients being split into two groups. One consists of those who are thought to lack agency (at least when suffering from an episode of illness), who cannot recover by their own efforts, and who are unlikely to be able to contribute significantly to their recovery. The other contains those who retain agency, could act differently, but choose not to. For example, patients with personality disorder are usually thought to retain agency (they could choose to do things differently), whereas those ill with affective disorders and psychoses are thought not to. Although, if asked, mental health professionals may agree that the situation is more nuanced, this ‘rule of thumb’ approach is widespread, and has negative consequences. Control, or agency, when assumed to be fully present, can make it difficult for professionals not to blame patients, since if you are responsible for your actions, and some of those actions harm yourself or others, you deserve to be blamed. This can lead to poor treatment and stigma from the belief that the unpleasant and harmful behaviours by the patient are a conscious choice.

Box 1 Dichotomising agency in the clinical situation

Helen has a family history of depression and self-harm. She has become more suicidal following the break-up of her marriage. She disclosed to her care coordinator that she had a rope and is intending to kill herself on her wedding anniversary. She is diagnosed with an exacerbation of chronic treatment resistant depression. She is admitted and placed on general observations.

Over the course of the next few weeks she ties ligatures around her neck and cuts herself with smuggled razors. As the perceived riskiness of her behaviour escalates, her level of observations are gradually increased. Staff find it difficult to tolerate her levels of distress and aggression.

Staff become split. Some feel she is severely depressed, and that she cannot help acting in this way. They spend increasing amounts of time with her. Others feel her problems are ‘behavioural’, by which they mean they are under her control; they wonder if she has a personality disorder, and advocate discharge.

## The place of agency in recovery

An all-or-nothing approach to agency also has an adverse effect on those deemed unable to exercise choice and control. The assumption is that change is beyond the power of the patient, and is in the hands of professionals, or in some cases down to chance. But symptoms (when they are behaviours, as they often are in psychiatry) and maintaining factors of a range of common psychiatric conditions include actions and omissions. Examples include addictions, eating disorders, depression and anxiety disorders, and the list could be expanded to include all conditions for which there is treatment which requires concordance as, if the patient decides not to take their medication, say, the omission will affect their condition and act as a maintaining factor. Actions and omissions that are either core features or maintaining factors of psychiatric disorders involve a degree of control, and thus recovery from these conditions requires the patient to have the motivation and will to change their behavior.^9^ An anxious patient who finds it difficult to leave the house may eventually recover simply by taking a selective serotonin reuptake inhibitor, but at some point they will have to take the difficult decision to go out of the front door, and the sooner they do (for example, as part of a treatment programme) the sooner they will recover. Telling a patient, or implying, that they have no control over their behaviours undermines their contribution to their recovery, and, if they conclude from this that they should cease their own efforts, may prevent recovery altogether.

## The wedge model of responsible agency

What is the alternative to the idea that patients are either subject to compulsion, and therefore entirely lacking in agency, or easily able to change their behaviours, but refusing to do so? It is the idea that control comes in degrees. This is a concept with which we are familiar when applied to ourselves, but have a tendency to forget in the clinical situation. Thus we find it easier not to shout at the kids when we are well rested and happy, more difficult when tired and stressed. The ability to exercise control (responsible agency) may be reduced by physical states like fatigue and pain, and emotional states like fear, anger and anxiety. In other words, it bears some relation to motivational and epistemic context;^8^ the ability to exercise control may vary with motivation, and with one's understanding of the situation and consequences – a smoker may stop when she becomes pregnant despite having previously attempted to do so without success.

Agency might also be impaired, to an extent, in a range of psychiatric disorders. Henderson lists ways in which this might happen ‘through a defect in consciousness, a change in mood, in perception, in the ability to think or the content of thought’.^4^ It may be more difficult for an agoraphobic to leave their house than it would be for you or me, for these reasons, but the impairment is one of degree, and although leaving the house may be difficult, it is not impossible. This understanding forms the basis of the behavioural treatment of agoraphobia.

The tendency to think of behaviour in some psychiatric disorders as compulsive (wholly without choice) is unsustainable also in view of the way people suffering from these disorders behave in practice. People with these problems quite commonly do change their behaviour. Some interventions bolster the ability to choose; for example, buprenorphine reduces the euphoric effects of opiates, and behavioural experimentation improves the ability to tolerate phobic stimuli. Psychiatric treatments change behaviours across a range of disorders and interventions.^10,11^

It also appears to be the case that behavioural changes become progressively easier as agency gradually increases. For example, the theory of behavioural activation for depression suggests that completing easier tasks leads to an improved ability to undertake more difficult tasks, possibly related to the impact of increasing self-efficacy on agency. This rationale is also seen in the graded exposure hierarchies used in the behavioural treatment of anxiety. In addition, experiments indicate that effortful practice appears to bolster willpower, the so-called ‘muscle model’ of the will.^12^

## Could retaining the idea of agency, and thus choice, invite stigma?

It is possible that widening the arena in which choice is considered a factor could subject those who suffer conditions that may be less subject to agency-related stigma, such as depression, to the additional stigma that those suffering disorders thought to be more choice-based, such as personality disorder, are subject to. Might professionals, and the public, have more difficulty feeling compassion if we reconsider the contribution made to mental disorders from patient choices?

This is possible, but is not a reason to retain an inaccurate approach to agency. Maintaining that a person has no control over a situation, when they appear to in fact retain some control, is not a viable solution to stigma, and the folk are probably not convinced by this anyway. Although people are generally willing to allow some slack to people who are ill, they are also sensitive to when the sick person appears to be ‘overstepping the mark’.^13^

## Ways of avoiding stigma when a degree of agency is retained

How might we mitigate any negative effect of acknowledging the place of will in the maintenance of mental disorder? ‘Responsibility without blame’ is a concept observed by a philosopher when visiting democratic therapeutic communities.^14^ Blame used in this context refers to affective blame, the negative feelings and attitudes that arise in another when someone is responsible for an action with a negative consequence. Pickard noticed that the staff of the therapeutic communities were able to retain the idea that patients with personality disorder were responsible for their decisions and actions – that they retained agency – while not engaging in blaming behaviours, or adopting a blaming attitude, that would be normal when those decisions and actions have negative consequences. She concluded that responsibility and blame can be separated, and should be for the purposes of good clinical care. Blame is usually countertherapeutic, and makes providing compassionate care more difficult, whereas the attribution of responsibility is essential both in motivating change, and in maintaining a mutually respectful therapeutic relationship (if your patient is not responsible for their apparent choices, you should treat them not as an equal moral being, but more like a child). Pickard thought that paying attention to the patient's personal history was one of the elements that make it possible to avoid blame, as this evokes compassion and empathy, which make affective blame less likely.

The acknowledgement of choice and control should not be allowed to affect treatment. This is already a problem, for example, when patients who have self-harmed are treated poorly in emergency departments.^15^ The solution to this is not to maintain the fiction that such behaviours are outside the patient's control, but to train professionals to act with compassion and care no matter the cause of the patient's distress.

## What are the practical implications of this approach?

If the exercise of free will is essential to recovery from mental disorders, we should treat people in such a way as to encourage the development of agency, to improve their capacity for control. It is possible to support patients through the difficult process of change, without moving to a paternalistic position in which change and recovery depends on us rather than the patient.^9^ Our approach to patients should thus be to acknowledge and bolster their power and agency in relation to their condition. In practical terms, when a patient tells us they cannot exercise control, such as to get out of bed when feeling depressed, it is helpful to regard them as able to exercise control, but to explore with them the degree to which this may be reduced, and the reasons for this. We should also work to avoid demoralising self-blame; for example, by emphasising that it is common for someone in their position to have these problems, that there are strategies for addressing it, and that it is okay to find it hard. It will be important to be circumspect in how this conceptualisation is used with regard to carers and relatives; the public is no less likely to dichotomise agency than professionals, and may react to the idea that agency is retained to a degree, by assuming that the patient is acting this way ‘on purpose’, a perennial problem in psychiatric disorder.

There is an additional point to note. As conditions such as obesity and addiction increasingly come to be seen as illnesses, or even diseases, people see themselves as less responsible and less able to change, with consequent increasing reliance on the efforts of professionals rather than themselves.

## Conclusion

Most mental disorders probably affect agency, making it more difficult to implement good choices. The extent to which this affects the patient will depend on both the nature and the severity of the disorder. Although it is probably true that people with even severe personality disorders retain agency much of the time to a greater degree than people with severe affective and psychotic disorders, agency is likely to be retained to some degree in all but a small proportion of patients with psychiatric disorders. Most psychiatric patients are able to contribute to their recovery through the exercise of their will. For this reason, treatment should emphasise the bolstering of control and willpower, which should include working to improve patients' understanding of their problems, and morale. In this way, we can avoid the twin mistakes of disempowering the patient by assuming they have no meaningful power to make choices that affect the course of their illness, and adopting a blaming attitude, which can allow the correct attribution of agency to detract from compassionate and energetic care.

## Footnotes

a. The term compulsion is used here in its general usage, meaning that a person is compelled to act, having no freedom to do otherwise. In psychiatric use, such as in obsessive–compulsive disorder, a compulsion can sometimes be resisted.

## About the author

**Steve Pearce,** Consultant Psychiatrist and Programme Director, Oxfordshire Complex Needs Service, Oxford Health NHS Foundation Trust, UK
